# The Spectrum of Autoimmune Thyroid Disease in the Short to Medium Term Following Interferon-*α* Therapy for Chronic Hepatitis C

**DOI:** 10.1155/2009/241786

**Published:** 2009-08-31

**Authors:** Huy A. Tran, Glenn E. M. Reeves

**Affiliations:** Hunter Area Pathology Service, Hunter Mail Region Centre, John Hunter Hospital, Locked Bag Number 1, Newcastle, NSW 2310, Australia

## Abstract

Autoimmune thyroid diseases are common manifestations of hepatitis C infection, exacerbated by interferon-based treatment. However, the occurrence and pattern of thyroid disease in the short/medium term following the completion of IFN-based therapy is relatively unknown and there are very few previous reports regarding the specific spectrum of autoimmune thyroid disease that may follow such therapy. We hereby report 3 cases which demonstrate the range of thyroid diseases that may occur following interferon therapy. The hypothesis advanced is that in the pathogenesis of these conditions there must be both triggering and sustaining mechanisms as thyroid diseases occur well outside the immediate effect window of pegylated interferon. This paper suggests the need to continue thyroid surveillance in IFN-treated HCV patients following the completion of therapy, perhaps for the first 6 months.

## 1. Clinical Case Notes

### 1.1. Case 1

A 53-year-old man underwent combination Ribavirin (RBV) and Interferon-*α*2*β* (IFN-*α*2*β*) for a total of 48 weeks because of his HCV genotype 1 and had achieved sustained virological response (SVR). During this time, he developed IFN-induced thyroiditis at 18 weeks with the classical biphasic thyrotoxic phase followed by hypothyroidism. The condition completely resolved by the 38th week, including normal thyrotropin (TSH), free tetra-iodothyronine (fT4), and free tri-iodothyronine (fT3). Thyroid hormone levels were followed monthly throughout the duration of treatment. There was no family or prior history of thyroid disease and antiviral therapy was continued despite his thyroid condition. He represented with general lethargy and weight loss of 3 kg two months *after* the completion of treatment for his chronic hepatitis C (HCV) infection. Clinical examination at this time showed sinus tachycardia at 101 beats per minute (bpm), blood pressure (BP) of 120/80 with no postural hypotension. There were peripheral stigmata of thyrotoxicosis but no signs of Graves' ophthalmopathy or dermopathy. No goitre was present. His TSH was undetectable (reference range (RR), 0.4–4.0 IU/L), fT4 19.8 (RR, 10.5–24.5 pmol/L) and fT3 8.9 (RR, 3.5–5.5 pmol/L). His thyroid pertechnetate uptake study was diffusely uniform and increased to 9% (RR, 3–8%), consistent with Graves' disease (GD). His TSH Stimulating Immunoglobulin (TSI) returned positive at 30 IU/L (RR, <10), his human TSH receptor antibody (hTRAB) was 15.8 IU/L (RR, <2). His antithyroglobulin (Tg), (RR, < 1 : 400), and antithyroperoxidase (TPO), (RR, < 1 : 400), levels were undetectable. A diagnosis of GD was made but the patient was reluctant to take medication as he was well with excellent exercise tolerance and thus treatment was withheld. Six weeks later, his fT3 level was 8.2 pmol/L and carbimazole was started. Three months later, he was clinically well and his fT3 level has normalised. Treatment was continued for 12 months during which his thyroid function tests were normal. His follow-up TSI and hTRAB antibodies had become undetectable at the end of treatment.

### 1.2. Case 2

A 56-year-old woman presented with T3-toxicosis 6 weeks following the completion of combination RBV and IFN-*α*2*β* for her HCV infection. She had undergone antiviral therapy over the previous 48 weeks for her HCV genotype 4 without any thyroid complications and had achieved SVR. There was no previous personal or family history of thyroid disease. As part of treatment protocols, her monthly thyroid function tests for the duration of treatment had been entirely normal. Four weeks after the completion of therapy, she began to notice mild dyspnoea on exertion, intermittent palpitation and heat intolerance. There were no other symptoms of thyrotoxicosis. Clinically, she appeared well with a regular pulse of 92 bpm, BP of 130/80. No goitre was detected nor were there any signs of thyrotoxicosis. Her TSH was undetectable, fT4 was 24.1 and fT3 8.9 pmol/L. Her thyroid uptake scan was reduced at 2% (RR, 3–8). The thyroid ultrasound was also normal in size and appearance; there was no evidence of nodularity but mild increase in vascularity. Her TSI, hTRAB, anti-Tg, and anti-TPO antibodies were not detectable. One week later, her T3-toxicosis persisted at 8.4 pmol/L. A diagnosis of IFN-induced thyroiditis was made and low dose propanolol was prescribed given her symptoms. She was followed closely with monthly TSH, fT4, and fT3 levels. Eight weeks later, she had entered into the hypothyroid phase with TSH of 54.6 IU/L, fT4 8.8, and fT3 2.3 pmol/L. As the patient remained free of any hypothyroid symptoms and given the expected recovery in thyroiditides, thyroxine therapy was withheld. At 16 weeks, her thyroid function had returned to normal. Propanolol was ceased and when last reviewed, the patient was in excellent health with ongoing normal thyroid function tests.

### 1.3. Case 3

A 45-year-old woman presented for a routine review 8 weeks following her failed therapy with IFN-*α*2*β* for her chronic HCV. She had been generally well with no significant previous medical history although there was a strong family history of thyroid disease in her family, with both her mother and grandmother experiencing thyroid diseases of undetermined nature, culminating in both requiring thyroxine supplement. Treatment for her HCV infection (genotype 1) was to be 48 weeks of RBV and IFN-*α*2*β*. However, after 24 weeks, there was no reduction in viral load and treatment was terminated. Her monthly thyroid function tests had been normal till then. System review on this occasion did not reveal any symptoms to suggest thyroid disease. Clinical examination at the review visit showed no signs of hypothyroidism. Her vital signs were satisfactory, with normal tendon reflexes and no goitre. Her routine TSH was found to be 48.0 IU/L with undetectable fT4 level. The follow-up thyroid ultrasound revealed the presence of a small atrophic gland with total volume of 6 mls (RR, 6–10) [1]. Her anti-Tg and anti-TPO titres were 1 : 256 and 1 : 512, respectively. The TSI and hTRAB were 11 IU/L and 11.7 IU/L respectively. Her thyroid uptake study was low at 3%. A diagnosis of autoimmune hypothyroidism was made and consequently, thyroxine was started. She was stabilised on 100 *μ*g of thyroxine daily. At six month review, her anti-Tg, anti-TPO, TSI and hTRAB antibodies were undetectable. In retrospect, the combination of high TSI and hTRAB titres with hypothyroidism and a low pertechnetate uptake study was consistent with the presence of thyrotropin blocking antibodies (TB-Ab). Based on this probable underlying mechanism for the hypothyroidism, thyroxine was ceased. The patient remained well and independent of thyroxine supplement in the following 12 months. Interferon-induced thyroiditis (in the hypothyroid phase) was unlikely given that there was no hyperthyroid phase. Functional TB-Ab assay would have been definitive but unfortunately was not available. 

**Figure 1:**
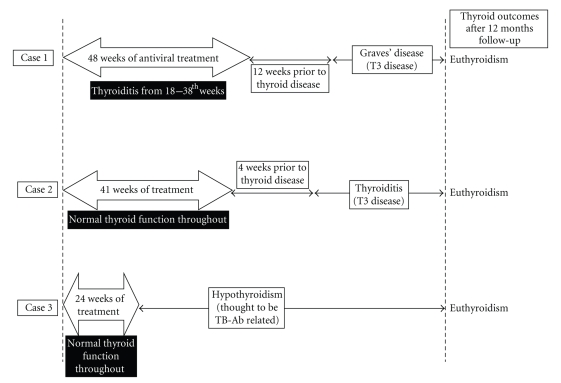
Schematic summaries of the cases and their final thyroid outcomes. The arrow bars indicate the duration of combination therapy with interferon-*α* and ribavirin. See text for detailed discussions.

 The clinical profiles of the three cases are represented schematically in Figure 1.

## 2. Discussion

The cases highlight the peculiar and fascinating spectrum of thyroid disease in the ensuing months following the completion of IFN-based therapy for chronic HCV infection. Our series demonstrates a wide spectrum of autoimmune thyroid diseases, ranging from GD to thyroiditis to profound subclinical hypothyroidism following IFN treatment. In case 1, the pattern includes GD following thyroiditis that occurred during the treatment phase. This unusual occurrence, referred to as “tri-phasic”, has been reported only once previously [2]. The predominance of T3 activity was also peculiar, without progressing to florid GD. In case 2, T3 thyroiditis occurred; this has not been described previously nor has it been described in this particular clinical setting. The clinical and biochemical behaviour is not very different from those arising de novo, expressing the classical biphasic response despite the negative antibody findings. Case 3 illustrates a complex mechanism of developing hypothyroidism in, presumably, a pre-existing abnormal thyroid gland. Despite normal TSH levels throughout the treatment duration, the development and resolution of hTRAB suggested the presence of TSH blocking antibody (TB-Ab) to account for the hypothyroidism, although this was never conclusively proven. The latter resolved, possibly following TBAB disappearance after the IFN effects had waned. In all 3 cases, no baseline antibody profiles were performed as they did not alter clinical management and thyroid status was monitored monthly during therapy. 

 There are only two previous studies documenting the long-term outcome of thyroid diseases but with IFN-*α* monotherapy, rather than combination therapy. Carella et al. [3] followed 114 patients for 6.2 years on average and did not find any overt thyroid dysfunction, only subclinical hypothyroidism in 12 cases at the end of therapy and 7 cases at the end of followup. Doi et al. [4] followed 17 patients to an average of 71 months in which there were 9 hyper- and 8 hypothyroid cases. No overt thyroiditis was observed, contrary to our current series. Tong et al. [5] studied the efficacy of *consensus-*IFN versus IFN-*α* (both in combination with RBV). This study included a follow-up period of 24 weeks posttreatment to coincide with the SVR review. Although both hypothyroidism and hyperthyroidism were included, no specific mention was made of their timing or characteristics. 

 The pathogenesis of this condition is in essence unknown but is probably distinct from the thyroid diseases arising during the course of IFN therapy [6, 7]. Clearly, a common denominator is recent exposure to IFN therapy for chronic HCV infection but it also clear that the process must have been perpetuated by additional factors. This persistence of the immune reaction up to 12 months after therapy had been observed previously in 3 patients [8]. All of these individuals achieved sustained virological response (SVR) whereas only 1 of our 3 cases did not. The exogenous IFN-*α* is believed to stimulate lymphocyte, macrophage and neutrophil function as well as increasing cytokine and chemokine concentrations, especially, interleukin-6 (IL-6) [8]. IFN-*α* induces MHC-II expression and probably CD40 expression on thyrocytes [9]. The latter results in an increased T-cell activation of the CD40 signalling pathway within the thyroid gland. This leads to an overexpression of intrathyroidal IL-6, synergising with pre-existing and circulating IL-6, in turn induces the development of thyroiditis. The high IL-6 is also thought to block TSH-mediated iodine uptake leading to the absent uptake scan [10]. Beside MHC-II, IFN also induces MHC-I expression on thyrocytes by way of IL-2 and chemokines [11], adding to the inflammatory response and the thyroiditis. IFN is also known to have a direct toxic effect on thyrocytes [10]. Furthermore, HCV particles have been found inside the thyrocytes [12] which could trigger and sustain an intracellular T-cell response and inflammation but this is unlikely assuming that there are no remaining intrathyroidal HCV particles in the presence of SVR. All these mechanisms are further amplified, exacerbated and maintained by the exogenous IFN-*α* therapy [13]. 

 Of recent interest is the function and place of regulatory T-cells (T_reg_) in HCV infection and IFN-associated thyroiditis. In HCV, the frequency of T_reg_ is high which directly suppresses T-cell overall function allowing the infection to persist [14]. In animal models, T_reg_ depletion induced lymphocytic infiltration of the thyroid leading to transient and/or permanent hypothyroidism [15]. Treatment with interferon-*β* in patients with multiple sclerosis results in an increased T_reg_ inhibitory capacity leading to the favourable outcome [16]. However, it is not known if this is the case for IFN-*α*. Furthermore, for the T-cell immunity to elicit the various immune thyroid disease, T_reg_ response should be dampened rather than enhanced to removs its inhibitory capacity and allow the T-cell helpers (T_H_) to initiate the autoimmunity process. 

**Figure 2:**
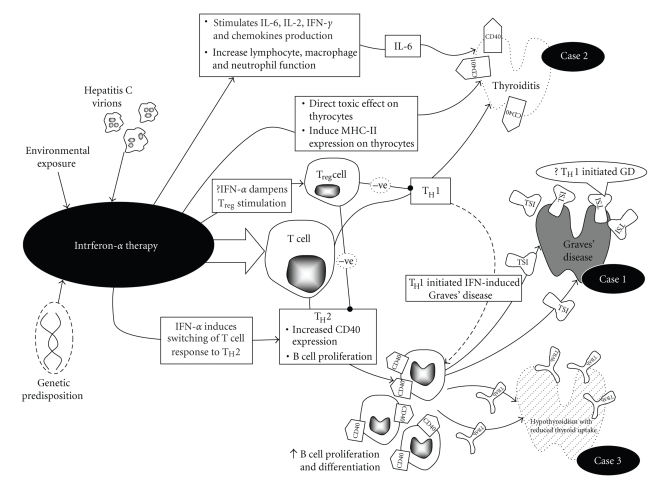
The proposed hypotheses for the development of the full spectrum of thyroid diseases after the completion of combination IFN-based therapy. IL-6: Interleukin-6; IFN: Interferon; MHC-II: major histocompatability complex-II; GD: Graves' disease; T_H_: T helper.

 The discussed immune mechanism above is thought to be under the auspices of the T_H_1 response. In GD, IFN is thought to induce or modulate switching of the T-cell response to T_H_2. This, in turn, stimulates B cell proliferation and differentiation under the influence of IL-6 and increased CD40 overexpression, resulting in an increase in TSI, simulating GD [9]. However, Nagayama et al. [17] suggested additionally that T_H_1 in itself might be a potential pathogenic mechanism in GD. Similarly, the apparent hypothyroidism is thought to be elicited by a similar mechanism but involving the alternative TB-Ab. There is no apparent reason to explain why the induced antibodies should be preferentially be stimulating, inhibitory, neutral or even a mixture. The pathogenesis is highly IFN dependent and resolves once the prolonged IFN effect has waned. This represents a potentially reversible pathway for hypothyroidism other than the hypothyroid phase of thyroiditis previously described [18]. The complex pathogenesis of this condition as currently understood has been summarised in Figure 2. 

It is well documented that hepatitis C infection is associated with an increase in endocrinopathies, especially autoimmune thyroid diseases, prior to and during therapy [7, 19], with a prevalence of 7–19% [20] depending on the studied population. Previous reports suggested that IFN therapy and female gender contribute to disease risk [20, 21]. Our previous published data suggested that thyroid surveillance is carried out monthly during treatment [7], although the frequency of surveillance remains contentious and the National Academy of Biochemistry is yet to recommend thyroid testing in this clinical scenario [22]. Three monthly TSH as suggested by Mandac et al. [19] may miss the entire diagnosis as thyroid functions have completely normalized in many cases. Both the British Society of Gastroenterology and the American Gastroenterological Association recognise the potential thyroid effect of IFN and recommend thyroid screening [23, 24]. However, only the former specifies that thyroid function testing is recommended at *each* treatment visit, rather than monthly. The National Institute of Health consensus statement on hepatitis C management surprisingly did not address this issue [25]. 

 To add to the complexity of the issue, it remains unknown if thyroid diseases are increased post-IFN therapy and if female gender is a risk factor, especially once this highly immunostimulatory virus has been eradicated. This is the first case series to explore the wide but probably incomplete spectrum of autoimmune thyroid dysfunction immediately following the completion of IFN-based treatment. This case series is small and the development of thyroid disease may arguably be incidental. However, the observation strongly suggests a persisting and disrupted immune system. These cases highlight the need for ongoing suspicion of thyroid disease and probable thyroid surveillance strategy, at least during the immediate 6–12 weeks following therapy completion and perhaps extending it to coincide with the time of SVR review at 6 months posttherapy followup.
